# *Announcement*: ALS Awareness Month — May 2017

**DOI:** 10.15585/mmwr.mm6616a6

**Published:** 2017-04-28

May is ALS Awareness Month, observed to raise awareness of and foster research for amyotrophic lateral sclerosis (ALS), also known as Lou Gehrig's disease. ALS is a progressive, fatal, neurodegenerative disorder of upper and lower motor neurons. The cause of ALS is not known, and no cure exists.

In October 2010, the Agency for Toxic Substances and Disease Registry (ATSDR) launched the congressionally mandated National ALS Registry (https://wwwn.cdc.gov/als/) to collect and analyze data regarding persons in the United States with ALS. The goals of the registry are to determine the incidence and prevalence of ALS, characterize the demographics of persons living with ALS, and examine possible risk factors for the disease.

In August 2016, ATSDR released its second prevalence report, which indicated an estimated 16,000 persons (5.0 per 100,000 population) were living with ALS in 2013. ALS remains more common among whites, males, non-Hispanics, and persons aged 60–69 years. The registry uses data from existing national databases, as well as the registry’s online system to track ALS cases. Online registrants can also take surveys regarding potential risk factors for the disease.

A new National ALS Biorepository allows researchers to request high-quality biologic specimens to study ALS. Both in-home and postmortem specimens are being collected from interested patients enrolled in the National ALS Registry. Epidemiologic data from patient surveys will be matched with patient specimens, making the biorepository a rich data source for ALS research.

ATSDR is collaborating with the ALS Association (http://www.als.org), the Muscular Dystrophy Association (https://www.mda.org), the Les Turner ALS foundation (http://www.lesturnerals.org), and other organizations to spread awareness about the National ALS Registry. Additional information is available at https://www.cdc.gov/als.

